# Potential benefits of an integrated military/civilian trauma system: experiences from two major regional conflicts

**DOI:** 10.1186/s13049-017-0360-6

**Published:** 2017-02-21

**Authors:** Jeffry L. Kashuk, Kobi Peleg, Elon Glassberg, Adi Givon, Irina Radomislensky, Yoram Kluger

**Affiliations:** 1 Assia Medical Group, Barzel 20, Tel Aviv, 96303 Israel; 2 Disaster Medicine Division, Faculty of Medicine, Tel-Aviv University, Tel-Aviv, Israel; 3 Israel Defense Forces, Jerusalem, Israel; 4 National Center for Trauma and Emergency Medicine Research, Gertner Institute for Epidemiology and Health Policy Research, Tel Hashomer, Israel; 5 Rambam Health Campus, Haifa, Israel

## Abstract

**Background:**

Although differences of opinion and controversies may arise, lessons learned from military conflicts often translate into improvements in triage, resuscitation strategies, and surgical technique. Our fully integrated national trauma system, providing care for both military and civilian casualties, necessitates close cooperation between all aspects of both sectors. We theorized that lessons learned from two regional conflicts over 8 years, with resultant improved triage, reduced hospital length of stay, and sustained low mortality would aid performance improvement and provide evidence of overall trauma system maturation.

**Methods:**

We performed an 8 year, retrospective analysis of the Israeli National Trauma Registry prospective data base for all casualties presenting to level 1 and 2 trauma centers nationwide during an earlier conflict (W1) (7/12/06-8/14/06) and sought to compare results to those of a more recent war(W2), (7/08/14-08/26/14), as well as to compare our results to non-war civilian morbidity and mortality during the same time frame. Of particular interest were: casualty distributions, injuries/ISS, patterns of evacuation/triage, hospital length of stay, and mortality.

**Results:**

Data on 919 war casualties was available for evaluation. Of 490 evacuated during W1, 341 (70%) were transferred to Level 1 centers, compared with 307 (72%) from the 429 casualties in W2. In W2, significantly more severe injuries (ISS ≥16) were evacuated directly to level 1 centers (42, 76% vs. 20, 43% respectively; *p =* 0.0007). W2 vs. W1 saw a significant increase in evacuations using helicopter (219,51% vs. 180,37%; *p <* 0.0001) and increase in ISS ≥16: (66; 15.5% vs. 55; 11%, *p =* 0.057). In W2 vs. W1, less late inter-hospital transfers occurred: (48, 11% vs. 149, 30%, *p <* 0.0001); and there was a reduction in admission ≥ 7 days (90,22%vs 154,32%, *p =* 0.0009). These results persisted in logistic regression analyses, when controlling for ISS..Mortality was not significantly changed either overall or for injures with ISS ≥ 16: (1.2%in W1 vs. 1.9% in W2, *p =* 0.59, 10.9% in W1 vs. 10.6% in W2, *p =* 1.0, respectively). When compared to civilian related, (non-war) mortality during the same 8 year time frame, overall mortality was unchanged (1.6% vs. 1.8%, *p =* 0.38), although there was a noteworthy significant decrease in mortality over time for ISS ≥ 16: 12.1 vs. 9.4 (*p =* 0.012), and a concomitant reduction in late inter-hospital transfers (9.8 vs. 7.5, *p <* 0.0001).

**Conclusion:**

Despite more severe injuries in the most recent regional conflict, there was increased direct triage via helicopter to level 1 centers, reduced inter-hospital transfers, reduced hospital length of stay, and persistent low mortality. Although further assessment is required, these data suggest that via ongoing cooperation in a culture of improved preparedness, an integrated military/civilian national trauma network has also positively impacted civilian results via reduced mortality in ISS ≥ 16 and reduced late inter-hospital transfers. These findings support continued maturation of the system as a whole.

## Background

Advances in civilian trauma care often parallel or follow similar experiences in military conflict [1–5]. Indeed, recent reports from casualties in Iraq and Afghanistan suggest that upwards of 95% of battlefield injuries have survived when prompt care and triage are implemented promptly, utilizing air transport to advanced level treatment facilities within the golden hour following initial trauma [6–9]. The concept of triage, or prioritization of wounded in an attempt to maximize survivors, was first introduced by the French in World War 1 [10]. After implementation of these techniques in the Korean and Vietnam conflicts, triage became standard practice world-wide in civilian trauma care [11]. Other major advancements commonly attributed to wartime experiences include: antibiotic use, blood banking techniques, use of wound adhesives, hemostatic bandages, tourniquet use, and hemostatic resuscitation.

Although Penicillin was first discovered by Fleming in 1928 [12], the antibiotic was not produced in large scale until World War 2 for use with casualties, following the discovery of fermentation processes to enable mass production in the 1940’s [13]. Similarly, around 1940, with the German invasion of Britain, perfection of blood banking techniques were accomplished by Drew which enabled separation of blood from plasma as well as transport and collection techniques which linked battlefields to hospitals [14, 15]. With improved blood availability, resuscitation techniques underwent changes as well. Improved crystalloid formations led to balanced salt solutions being used as initial and continuing resuscitation techniques through the Viet Nam conflict, contributing to the early recognition of the “Da Nang Lung = ARDS”, and compartment syndromes associated with over-zealous resuscitation [16, 17]. These concerns clearly influenced the evolution of the modern concept of hemostatic resuscitation, with use of minimal crystalloid, whole blood when available, and component therapy of packed cells, plasma, and platelets in ratios approximating 1:1 [18–22]. These concepts have been translated to civilian practice with improved survival and reduced morbidity [23–26].

During the Viet Nam War (1954–1975), wound adhesives first were used in the field as a spray form [27], later evolving into stronger bonds with more effective use in recent years [27, 28]. During operation Desert Storm, hemostatic bandages such as Quick clot, containing kaolin, were aggressively used in an attempt to accomplish temporary bleeding control in the field The effectiveness of this approach was recognized by the US Army Surgeon General, who required that all soldiers serving carry at least one hemostatic bandage [29, 30].

While the use of the tourniquet is a simple technique dating back to antiquity, a recent simple but genius concept, the one handed CAT, or Combat-Application-Tourniquet, consisting of a band that slips onto an extremity and easily twists to control blood flow, has significantly impacted limb salvage rates in recent conflicts and has become standard practice with civilian first responders [31]. Despite enthusiasm for this simple but lifesaving action, surprisingly, the technique is not yet fully accepted as part of the primary resuscitation armamentarium for first responders nationally in the United States [32].

Clearly, most all of the advances were either conceived or implemented during military conflicts over the years due to the urgency at hand. Translation of these concepts to civilian practice, however, may not always occur promptly or efficiently, although, in the current internet age with improved communication and cooperation, the process of clinical adaption has been clearly impacted.

Disaster and Mass casualty triage implementation probably represents the single most urgent and essential category world—wide where translation of military lessons learned to civilian practice is essential and in fact, due to the fact that terror is occurring on our city streets with increasing frequency, rapid transfer of knowledge, experience, and lessons learned has become an urgent priority.

The nation of Israel presents a unique model for assessing the relationship between military and civilian sectors of care. Although the nation was established in 1948, the modern trauma system was implemented over the past 20 years. As a small nation, about the size of New Jersey, transport times are short. While Israel does not have a dedicated military hospital system, trauma care for casualties in our military conflicts is provided in civilian medical centers nation-wide. Clearly, lessons learned during military conflicts are immediate and directly impact civilian care.

Since the Israeli trauma system is dependent on a fully integrated military and civilian plan, we hypothesized that performance improvement assessment of both facets of the system would reflect such cooperation.. Accordingly, we sought to analyze and compare triage, hospital, as well as morbidity and mortality data over the past 8 years from two recent military conflicts.

## Methods

The Israeli Trauma Registry, managed by the Israel Center for Trauma and Emergency Medicine Research at The Gertner Institute, was established as a central repository for all military as well as civilian trauma data in the state of Israel in 1995, maintaining a continuous prospective data base. The registry includes casualties who were admitted to or died in the hospital. It does not include those that were discharged from hospital after emergency department treatment or dead on arrival or dead at the scene.

In the current evaluation, we performed an 8 year, retrospective analysis of all casualties arriving at level 1 and 2 trauma centers injured in two most significant regional conflicts: The Second Lebanon War (W1) (7/12/06-8/14/06), and the Protective Edge War (W2)(7/08/14-8/26/14). The Israeli statewide trauma system consists of 6 level 1 and 14 level 2 trauma centers. All trauma centers in Israel accept patients from military conflicts, although clearly, level 1 trauma centers in closest proximity to regional conflicts have traditionally received the bulk of admissions. In the current study, data from W1 was available from 6 central level 1 facilities and 5 regional level 2 facilities. During W2, data was available from 6 central level 1 facilities and 11 regional level 2 facilities. Furthermore, we sought to compare triage patterns and mortality rates in the military sector to our civilian data base during the same time frame. The civilian data included non-war casualties that were hospitalized during two 8 month periods: January-August 2006 and same months in 2014. Principle data of interest included: casualty distributions, injuries/ISS, patterns of evacuation/triage, hospital length of stay, and mortality.

Statistical analysis was performed using SAS V 9.4 statistical software. An alpha criterion for statistical significance was set at .05. For examining the association between categorical variables a chi-squared test or Fisher exact test was used. Multivariate logistic regression analysis was performed to examine the probability of hospital admission ≥7 days and hospital transfers when the specific conflict and ISS were taken into account.

## Results

Data on 919 casualties was available for evaluation. The results of all transfers by level of trauma center are described in Table 1. Of 490 evacuated during W1, 341 (70%) were transferred to Level 1 centers, while the remainder (149, 30%) were transferred to level 2 centers. In W2, 429 total casualties were distributed to Level 1 (307, 72%), and level 2 (122,28%) centers. In W2, significantly more severe injuries (ISS > 16) were evacuated directly to level 1 centers (42, 76% vs. 20,43% respectively; *p =* 0.0007). Table 2 lists the mode of transfer of patients to the trauma facilities, comparing helicopter vs. ground transport. W2 vs. W1 saw a significant increase in evacuations using helicopter (219, 51% vs. 180, 37%; *p <* 0.0001). In W2 vs. W1, less late inter-hospital transfers occurred: (48, 11% vs. 149, 30%, *p <* 0.0001) Table 3 lists the results of injury severity by patient transfer, as well as the subsequent duration of hospitalization. In comparing W1 to W2, there was a significant increase in ISS ≥ 16: (66; 15.5% vs. 55; 11%, *p =* 0.057), while there was a reduction in admission ≥ 7 days (90,22%vs 154,32%, *p =* 0.0009). Table 4 demonstrates that in logistic regression analyses, when controlling for ISS, these results persisted: admission ≥7 days: (OR = 2.3, 1.63–3.3 95% CI, *p <* 0.0001); late hospital transfers: (OR = 3.9, 2.71–5.68 95% CI, *p <* 0.0001). Table 5 shows the results comparing overall mortality in the two conflicts. For patients arriving with vital signs to the ED, mortality was not significantly changed either overall or for injures with ISS ≥16: (1.2%in W1 vs. 1.9% in W2, *p =* 0.59, 10.9% in W1 vs. 10.6% in W2, *p =* 1.0, respectively). When these results were compared to civilian related, (non-war) mortality during the same 8 year time frame, (Table 6) overall results were unchanged (1.6% vs. 1.8%, *p =* 0.38), although there was a noteworthy significant decrease in mortality over time for ISS ≥16: 12.1 vs. 9.4 (*p =* 0.012), and a concomitant reduction in late inter-hospital transfers (9.8 vs. 7.5, *p <* 0.0001).

**Table 1:** Characteristics of patients by level of trauma center and severity of injuries in two wars

Variable	W1 *N =* 490% (n)	W2 *N =* 429% (n)	*p-*value*
Trauma center			.56
Level 1	69.6 (341)	71.6 (307)	
Level 2	30.4 (149)	28.4 (122)	
ISS >16 Level 1	*n =* 46	*n =* 55	.0007
Directly to Level 1	43.5 (20)	76.4 (42)	
Secondary Transfer to Level 1	56.5 (26)	23.6 (13)	

*Fisher exact test

**Table 2:** Helicopter evacuation and patient transfer in two wars

Variable	W1 *N =* 490% (n)	W2 *N =* 429% (n)	*p-*value*
Evacuation type			<.0001
Helicopter	36.7 (180)	51.1 (219)	
Ground transport	63.3 (310)	48.9 (210)	
Patient Transfer			<.0001
Transferred	30.4 (149)	11.2 (48)	
Non transferred	69.6 (341)	88.8 (381)	

*Fisher exact test

**Table 3:** Injury severity and admission >7 days in two wars

Variable	W1 *N =* 490% (n)	W2 *N =* 429% (n)	*p-*value*
ISS			.057
1–14	88.8 (435)	84.5 (360)	
> 16	11.2 (55)	15.5 (66)	
LOS			.0009
0–6 days	68.4 (333)	78.2 (323)	
> 7 days	31.6 (154)	21.8 (90)	

*LOS* Length Of Stay

*Fisher exact test

**Table 4:** Logistic regression models^a^ W1 vs. W2

Dependent Variable	OR (95% CI)	*P* value	Concordance Index C
LOS >7d	2.3 (1.63,3.30)	<.0001	0.774
Transferred	3.9 (2.71,5.68)	<.0001	0.693

^a^Adjusted ISS (1–8, 9–14, 16–24, >25)

**Table 5:** In-hospital mortality in two wars compared to 8 month non-war (civilian injuries) time frame

Variable	W1 *N =* 490% (n)	W2 *N =* 429% (n)	*p-*value^*^	1-8/2006 *N =* 15,945% (n)	1-8/2014 *N =* 17,931% (n)	*p-*value^*^
In-hospital Mortality	1.2 (6)	1.9 (8)	.59	1.6 (260)	1.8 (315)	.38
Transferred	30.4 (149)	11.2 (48)	<.0001	9.8 (1,557)	7.5 (1,340)	<.0001
ISS > 16	*n =* 55	*n =* 66		*n =* 1,600	*n =* 2,014	
In-hospital Mortality	10.9 (6)	10.6 (7)	1.0	12.1 (193)	9.4 (190)	.012

^*^Fisher exact test

**Table 6:** Civilian and military trauma in two wars and in 8 month non-war period in 8 year time frame

Variable	W1 *N =* 490% (n)	W2 *N =* 429% (n)	*p-*value*	1-8/2006 *N =* 15,945% (n)	1-8/2014 *N =* 17,931% (n)	*p-*value*
Population group			<.0001			<.0001
Citizen/Other	25.7 (126)	13.3 (57)		97.3 (15,509)	98.2 (17,616)	
Soldier	74.3 (364)	86.7 (372)		2.7 (436)	1.8 (315)	

*Fisher exact test

## Discussion

Many of the most significant advances in trauma care world-wide have occurred as a direct result of lessons learned during military conflicts. Major advancements commonly attributed to wartime experiences include: antibiotic use, blood banking techniques, use of wound adhesives, hemostatic bandages, tourniquets, hemostatic resuscitation, and vascular shunts [33]. Furthermore, although triage in military conflict may have traditionally involve principles quite different from civilian scenarios [34, 35], the current terror wave facing our cities world-wide have required a reassessment of triage techniques even in the civilian sector [34–41]. Such challenges have necessitated improved military-civilian cooperation and rapid implementation of new techniques to deal with the mass casualty challenges at hand. Despite this, current evidence suggests that a significant lag time still exists for acceptance of military principles in the civilian sector, due to logistic differences, established civilian vs. military protocol variety, as well as local and regional differences in trauma care within the civilian sector.

The Israeli national trauma system is a fully integrated and coordinated program where all civilian and military casualties are triaged to the closest trauma center. Due to Israel’s small size, transport times tend to be quite brief. In comparing our results from two regional wars we noted several significant improvements which occurred over the eight year time frame of study. Despite more severe injuries in our most recent regional conflict, we noted increased direct helicopter transfers to level 1 centers, with concomitant reduced inter-hospital transfers. These results support improved efficiency of military triage. Of note, these findings persisted in logistic regression analysis when controlling for ISS. Efficient and effective triage has been cited by others as an important contributor to improved outcomes [39–41], and our results of reduced hospital length of stay and low mortality, corroborate these findings.

Recognizing the unique nature of a fully integrated model, we further sought to compare civilian, non-military mortality in the trauma system to the current results from two military conflicts. These results showed a significant nationwide decrease in mortality for ISS ≥16 during the similar time frame, as well as a significant reduction in late inter-hospital transfers.

While the current data may provide evidence of overall trauma system maturation, there are limitations to this study. In addition to the known limitations of a retrospective analysis, several important logistic, tactical, and other differences in the operational settings between the two conflicts should be recognized. W1, which occurred in Northern Israel along the Lebanon border, involved tens of thousands of reservists, with several divisions maneuvering into south Lebanon. The medical support for these troops was based on battalion aid stations, manned with two physicians attached to each regiment. Of note, the civilian population of Northern Israel was heavily bombed by missiles, resulting in > 2000 civilian casualties who required parallel triage to the same civilian hospitals. Field dangers, such as the presence of anti-tank and surface to air missiles resulted in delayed extractions and evacuations. Accordingly, the average evacuation of urgent casualties was approximately three hours [41], and the vast majority of the severely wounded were air-evacuated via helicopters staffed with physicians and paramedics capable of performing life—saving interventions including blood administration. W2, which occurred in Southern Israel, involved almost exclusively active duty forces involving very little maneuvering. Of all casualties, approximately 1/3 were civilians injured as a result of rockets and mortar launched towards surrounding towns. Medical support involved advanced life support (physician or a paramedic), as an integral part of the combat platoon, as well as the battalion aid stations. Certainly lessons learned from W1, such as shorter evacuation routes, varied evacuation platforms, and close air support certainly contributed to significantly shorter evacuation times [42, 43]. Furthermore, additional factors which potentially contributed to low case fatality rates in W2 included the availability of hemostatic dressings, advanced tourniquets and the administration of Freeze Dried Plasma as the resuscitation fluid of choice. Lastly, the geographic proximity to the civilian trauma centers in the south and center of Israel likely contributed to the shorter evacuation times.

## Conclusion

Although in the past, military and civilian trauma systems may have operated rather independently due to significant differences in logistics, as well as implementation of protocols, current challenges world-wide in the current terror epidemic show that improved cooperation and rapid implementation of lessons learned between sectors is certainly in the best interests of our patients and provides opportunities for growth and integration.

The Israeli model of unified military and civilian trauma care, which promotes a culture of cooperation and preparedness, suggests that even in trauma systems where more extensive separation is present, efforts towards closer cooperation are certainly beneficial to all.
